# A new measurement of an indirect measure of condom use and its relationships with barriers

**DOI:** 10.1080/17290376.2017.1375970

**Published:** 2017-09-13

**Authors:** Einav Levy, Yori Gidron, Benjamin O. Olley

**Affiliations:** ^a^ MPH, is a PhD Candidate at Faculty of Medicine and Pharmacy, The Free University of Brussels (VUB), Brussels, Belgium; ^b^ PhD, is a Professor at Faculty of Medicine and Pharmacy, The Free University of Brussels (VUB), Brussels, Belgium; ^c^ SCALab, Lille 3 University, Lille, France; ^d^ PhD, is an Associate Professor in Clinical & Health Psychology, Department of Psychology, University of Ibadan, Ibadan, Nigeria

**Keywords:** condom use, barriers, social desirability, assessment, measure, Utilisation du préservatif, Obstacle, désirabilite sociale, évaluation, mesure

## Abstract

One of the challenges facing researchers in the domain of human immunodeficiency virus prevention is the assessment of condom use in an unbiased self-reported manner. The current study presents the development and preliminary validation of an indirect condom use test (I-CUTE), designed to assess condom use tendencies and to overcome self-report biases. Two samples were included using correlational designs. In sample 1, 88 students from European university completed the I-CUTE with questionnaires of condom use barriers, social desirability, and condom use negotiation self-efficacy. In sample 2, 212 students from sub-Saharan universities completed the I-CUTE with questionnaires of condom use barriers and knowledge. The I-CUTE included 17 pictures of human figures in relation to condom use, where participants had to choose one of the four a-priori given sentences reflecting the figures’ thoughts. This represented a semi-projective, yet standardized test. In sample 1, I-CUTE scores were inversely related to barriers, positively correlated with condom use negotiation self-efficacy and unrelated to social desirability. In sample 2, I-CUTE scores were inversely related to barriers and unrelated to knowledge scores. In a multiple regression, condom use barriers had a unique contribution to explaining variance in I-CUTE scores, beyond the contribution of background variables and knowledge. These results support the preliminary reliability and validity of the I-CUTE tool in a variety of cultures, and reveal its lack of bias by social desirability and the importance of condom use barriers in condom use tendencies.

## Résumé

Un des défis auxquels les scientifiques dans le domaine de la prévention du VIH sont confrontés, est l’évaluation de l’utilisation du préservatif de manière autodéclarée non biaisée. L’étude actuelle présente le développement et la validation préliminaire d’un test indirect d’utilisation du préservatif (I-CUTE), conçu pour évaluer les tendances de l’utilisation du préservatif et pour surmonter les biais de l’auto-évaluation. Deux échantillons ont été inclus en utilisant des concepts corrélationnels. Dans le premier échantillon, 88 étudiants d'universités européennes ont complété l’I-CUTE au moyen de questionnaires sur les entraves à l’utilisation du préservatif, la désirabilité sociale, et l’auto-efficacité lors de la négociation sur l’utilisation du préservatif. Dans le deuxième échantillon, 212 étudiants d'universités Sub-Sahariennes ont effectué l’I-CUTE au moyen de questionnaires sur la connaissance de l’utilisation du préservatif et les entraves y afférent. L’I-CUTE incluait 17 photos de figures humaines en relation avec l’utilisation du préservatif, et les répondants devaient choisir parmi quatre phrases préalablement données la phrase qui reflètait au mieux les pensées des figures. Cela représentait un test semi-projectif, bien que standardisé. Dans le premier échantillon, les scores de l’I-CUTE étaient inversement liés aux entraves. Il y avait une corrélation positive entre les scores et l’auto-efficacité de la négociation sur l’utilisation du préservatif, et il n’y avait pas de rapport avec la désirabilité sociale. Dans le deuxième échantillon, les scores de l’I-CUTE étaient inversement liés aux entraves et sans aucun rapport avec les scores sur la connaissance. Dans une régression multiple, les entraves à l’utilisation du préservatif présentaient une contribution unique pour expliquer la variance dans les résultats de l’I-CUTE, bien au-delà de la contribution des variables de l’environnement et la connaissance. Ces résultats confirment la fiabilité préliminaire et la validité de l’outil I-CUTE dans une variété de cultures. Ils attestent l'absence de biais par la désirabilité sociale et la pertinence des entraves à l’utilisation du préservatif dans les tendances de l’utilisation de celui-ci

## Introduction

### The importance of condom use

Studies show that persistent condom use reduces the risk of contracting human immunodeficiency virus (HIV) (Mansergh, Herbst, Mimiaga, & Holman, 2015; Ramjee, Abbai, & Naidoo, 2015; Smith, Herbst, Zhang, & Rose, 2015; Weller & Davis-Beaty, 2002); hence, this is a major way to prevent HIV, worldwide. However, the precise prevalence of condom use is hard to determine since studies differ in their method of assessment, sampling, culture, age, gender, risk group, and epoch, amongst other factors. For example, over 66 studies conducted between 2003 and 2005, the prevalence of condom use in Chinese men who have sex with men was 61.6%. A systematic review done in China found that persistent condom use was reported by 36.3% of participants (Chow, Wilson, & Zhang, 2012). Yet another study shows that 57–59% of young men and 48% of young women report condom use in their most recent intercourse in Young South Africans (Setsuko, Pettifor, Lee, Coates, & Rees, 2007), and that only 24% of sexually active female participants had ever used a condom (Da Cruz, 1999). These discrepant findings could also result from other reasons, beyond demographic factors, as elaborated in the following section.

### Factors associated with condom use

Multiple factors have been associated with condom use and include demographic factors such as marital status, income (Janepanish, Dancy, & Park, 2011) and ethnicity, interpersonal relation factors such as having a steady partner (Abraham, Sheeran, & Henderson, 2014; Devries, Free, & Jategaonkar, 2007), and communication factors such as condom use negotiation self-efficacy and fear (Crosby et al., 2013). Another possible determinant of condom use is knowledge about the health risks when not using condoms. This is in line with the health belief model (Rosenstock, 1974). Yet, studies show that educational interventions aimed at increasing knowledge alone have little impact or statistically non-significant effects on condom use (Gallant & Maticka-Tyndale, 2004).

In contrast, several social cognitive factors such as perceived risk (Gallagher et al., 2014), knowledge of one's HIV status (Cherutich et al., 2012), subjective norms about significant others’ opinions concerning condoms, and perceived control and intention (Brüll, Ruiter, Wiers, & Kok, 2016; Janepanish et al., 2011) have also been found to be related to condom use. These social cognitive factors are derived from the theory of planned behavior (Ajzen, 1991). Such cognitive factors can be targets of interventions for increasing condom use beyond increasing knowledge alone.

Another group of specific social cognitive factors, which can influence the rate of condom use, refers to barriers concerning their use, in line with the health belief model. There have been several studies examining barriers against condom use. These barriers also differ as a function of gender, age, and culture. For example, one study in Mumbai, India, found that lack of privacy in stores and social stigma were most frequently indicated as barriers to condom use (Roth, Krishnan, & Bunch, 2001). Further barriers found were lack of trust in the reliability of condoms to protect against the disease (Mash, Mash, & De Villiers, 2010), reduced pleasure, perceived and actual physical side effects, and lack of information (Versteeg & Murray, 2008).

### Assessment of condom use

Beyond these social cultural and cognitive factors, one factor influencing the rate of documented condom use is its method of assessment. Several methods for assessing condom use exist, which relate to knowledge, attitude and skills, most of which are self-report (Fonner, Kennedy, O’Reilly, & Sweat, 2014). These include the Condom Use Skills Checklist, which consists of 17 items (Stanton et al., 2009), and another self-report questionnaire, which consists of six domains (Setsuko et al., 2007). Additional measures such as the Measurement of Observed Condom Use Skills were also developed for this purpose (Lindenmann & Brigham, 2003). The latter is an important advancement compared to others since it requires participants to demonstrate behaviorally their knowledge of using a condom. However, such a measure may not be feasible for mass testing in large populations. Furthermore, this behavioral measure does not assess the actual use of condoms in past intercourses, but only practical knowledge. Some investigators tried to validate such measures with biological markers (Prostate-Specific Antigen – PSA, in the vagina), yet found no relationships between them (Gallo et al., 2010). This could stem from the limitations of self-reported tools or since PSA levels may change due to multiple reasons other than sexual activity alone.

Most of the above-mentioned instruments for assessing condom use were self-report. However, using explicit self-report tools is limited due to methodological issues such as recall biases and presentation biases. Furthermore, health behaviors including condom use are affected by explicit (Deliberate cognitions and intentions) and implicit processes (Impulsiveness, sexual drive), in accordance with the Dual System Model (Hofmann, Friese, & Strack, 2009). The influence of such implicit processes may require a different method for assessing condom use, including more indirect methods. One example of an implicit test is the Implicit Association Test (IAT). The IAT has been used to identify predictors of condom use such as implicit attitudes toward condoms (Sakaluk & Gillath, 2016).

However, the IAT tool demands accessibility to technology and was developed according to certain cultural concepts. In contrast, most of the countries with high rates of HIV are the same countries with more limited accessibility to technology and are subject to cultural differences, which might affect the reliability and the use of the IAT in such cultures. Alternative, ‘lower tech’ yet indirect, reliable, and valid methods for assessing condom use are needed. The aim of the present study was to address this gap.

The Rosenzweig Picture Frustration Test (RPFT) is a tool that addresses these limitations since it is pictorial and requires no technology (Rosenzweig, 1978). In the RPFT, the participant is asked to estimate what a character in a scenario would think or do, thus, projecting his/her own thoughts onto the character. Originally, the investigator interpreted the response of the participant, a process which can be biased and reduce the reliability of the test.

### Purpose of study and hypostheses

The present study adopted the methodology of the RPFT when it comes to using the pictures and projective method. However, unlike the RPFT, we developed a tool that provided a-priori scaled answers, thus, removing the need for interpreting participants’ responses by the investigator. The aim of the present study was to develop and preliminarily validate such a tool called the indirect condom use test (I-CUTE). The I-CUTE aims to indirectly assess condom use tendencies. This study wished to examine the correlation between scores of this new instrument and other existing ones, by testing its convergent and divergent validity. Specifically, we hypothesized that the I-CUTE would have a negative correlation with a measure of condom use barriers, supporting its convergent validity. Moreover, we expected that the I-CUTE scores would be unrelated to social desirability, supporting its divergent validity. Furthermore, we examined the contribution of barriers concerning condoms in explaining variance in condom use tendencies assessed by the I-CUTE, independent of background variables and social desirability. Finally, we wished to compare the relative importance of barriers versus knowledge about HIV in relation to condom use tendencies. This was done by testing the unique importance of barriers concerning condom use, beyond the contribution of participants’ knowledge.

## Method

### Participants

In this study, two samples were included. In sample 1, a total of 88 students from a Belgian University and students from Ibadan University were included. This study was approved by the ethics board of University of Ibadan, Nigeria, and by the ethics board of Hoogschool Universiteit Brussels and was given as part of learning research methods and topics in health psychology and job stress. Their mean (SD) age was 21.84 (2.38) years. In sample 2, 212 students of agriculture and medicine in universities from Burkina Faso, Kenya, Uganda, Zambia, Tanzania, Rwanda, Ivory Coast, Nigeria, and Cameroun voluntarily took part. These students were taking part in an educational program in another country from which approval for the study was obtained. The population was chosen according to the participants’ age, risk of multiple partners, and its variety of origins. The participants’ mean (SD) age was 23.91 (3.63) years. In both samples, scales were completed anonymously.

### Measures

#### Demographic and background information

This included age, gender, having a partner, having sexual relations with the partner, and an explicit question directly inquiring about condom use in the past month. In relation to having a partner, this was assessed with a simple yes/no question in sample 1. In sample 2, we assessed this issue in detail by including four response categories: no partner, partner without sexual relations, several partners with sexual relations, and one partner with sexual relations.

#### Assessment of condom use tendency

This was done by the I-CUTE, whose development was the primary focus of this study. Using the RPFT paradigm, we initially included ten pictures depicting people either in intimate proximity (non-erotic) or with a condom. Under each picture, there was a question asking the participant what one of the characters *in the picture* was thinking or doing, reflecting the projective element of the RPFT. To answer that, participants had to choose one of four responses, which reflected their own tendencies to use condoms from low to high. In some pictures, the responses were from high to low, to avoid response sets. The indirect nature of the I-CUTE was manifested by asking the participant what was the thought or conclusion of a figure in the picture and not what the participant him/herself thinking. The scenarios were chosen to reflect issues pertinent to condom use, and included attitudes and awareness to health risks, relationships, commitment, and trust (Setsuko et al., 2007). These issues were seen as representing the broader contexts in which condoms are used. In order to adjust the pictures to a variety of origins and cultures, the chosen pictures were either vague, showing shadows of figures or mixing dark skin and white skin figures.

**Fig. 1.**
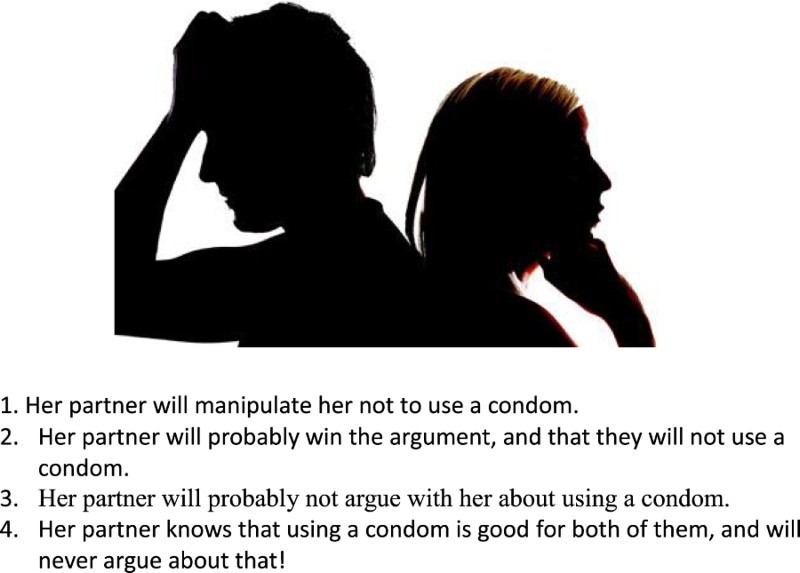
She knows that.

**Fig. 2.**
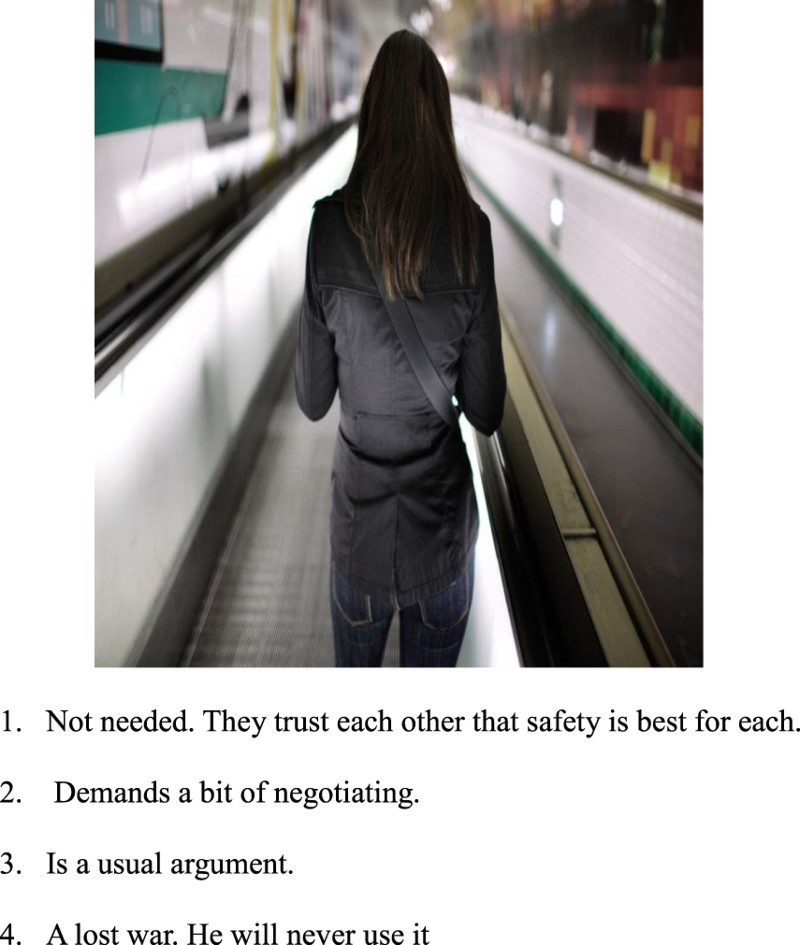
Asking her partner to use a condom is.

Figs. 1 and 2 show two examples of the pictorial test.

1. She knows that?
2. Asking her partner to use a condom is:

Higher scores reflect a high (indirect) tendency to use condoms. In a pilot study which included 10 items, initial Cronbach's Alpha was considerably low (alpha = 0.338). In order to increase the internal reliability, we removed three items which were found to decrease reliability. This led to an improved internal reliability of 0.505. However, since this was still below the acceptable reliability level of 0.7, we attempted to improve and clarify the phrasing of the text in the scenarios.

Further analysis of the items which reduced the reliability showed that the pictures in those items were too vivid. This might have caused the low reliability due to reducing the projective aspect of the test. Furthermore, 10 items may have been insufficient for achieving an adequate reliability, using a semi-projective technique. We thus increased the number of items to 20 instead of 10. In addition, we verified that each picture and its responses reflected one theme only (e.g. health education). In the 20 items test, we added pictures that were ambiguous in order to increase the projective element and thus, try to improve the reliability of the test.

Further analysis showed that three additional items were decreasing the internal reliability of the scale (Cronbach's Alpha, <0.7). Thus, these three items were removed. This 17-items scale reflects the final I-CUTE on which the following analyses are based.

Finally, though the I-CUTE is intended to both genders, we phrased the items and the scenarios’ contents primarily for female participants. This was done since past studies showed that women with high level of relationship power were five times as likely as women with low levels, to report consistent condom use. A proven strong association between the relationship power and consistency of using condoms emphasizes the key role of women's self-efficacy in safer sex decision-making (Bauermeister, Hickok, Meadowbrooke, Veinot, & Loveluck, 2014; Pulerwitz, Amaro, Jong, Gortmaker, & Rudd, 2002). These findings which underscore the major importance of women's ability to persuade their partners to use condom directed the structure of the I-CUTE to address the items to the female gender relatively more than to the male gender. Nevertheless, the following validation was done on both genders to increase the generalizability of the I-CUTE.

#### Condom use negotiation self-efficacy scale

An explicit four-items scale that measures one's assertiveness to persuade the partner to use a condom was used (Shaweno & Tekletsadik, 2013) in sample 1. In the present study, the internal reliability was Cronbach's Alpha = 0.72. An example of a question was ‘Do you feel confident in your ability to persuade partners to use condoms before sex started?’

#### Condom barriers scale

An additional explicit test was the condom barrier scale (Lawrence et al., 1999). This scale was developed to assess the level of barriers a person has concerning condom use. In sample 1, the internal reliability of this scale was high (Cronbach's alpha = 0.92). In sample 2, the internal reliability of this scale was high as well (Cronbach's alpha = 0.90). Each item is rated from ‘Strongly agree’ to ‘Strongly disagree’. An example of an item is: ‘Condoms don't feel good.’ In the present study, we reversed the scoring such that higher scores reflected greater barriers concerning condoms.

#### Social desirability scale (SDS-17) (Stober, 2001)

This scale measures the tendency to describe ones’ behavior in relation to perceived values in ones’ society. In the present study, this scale was only used in sample 1. Its internal reliability was Cronbach's Alpha = .63.

#### Knowledge test

This test included 10 questions with true/false responses. The number of correct responses was summed to yield the knowledge score. An example for a question is: ‘You can put the condom on, any time before the ejaculation, even at the last seconds.’

These 10 questions were obtained from various HIV/AIDS knowledge tests or online sites with educational information (AIDS Response, 2016; AIDS.gov, 2016; Carey & Schroder, 2002; Center for Disease Control and Prevention, 2016; Stanton et al., 2009).

### Design and procedure

The present study used a quantitative correlational design to validate the newly developed tool. All questionnaires were administrated in the following order: 1. I-cute, 2. Condom negotiation self-efficacy questionnaire (in both of the samples), 3. Condom Barriers Scale, and 4. Social desirability questionnaire (in both of the samples). We administrated the questionnaires in this order to reduce any possible biases from explicit measures affecting the I-CUTE, in order to increase the projective element of the indirect test.

## Statistical analysis

We conducted tests of internal reliability (including corrected item-total correlation tests) to identify items that reduced the reliability of the newly developed I-CUTE. To test its convergent validity, we tested the correlation between the I-CUTE scores and the condom negotiation self-efficacy scale and the condom barriers scale. In sample 1, we examined the correlation between the I-CUTE and the social desirability test, to test the divergent validity of the I-CUTE. We compared the relative importance of knowledge versus barriers in condom use tendencies (I-CUTE) by examining their inter-correlations. Finally, we also tested the unique contribution of barriers in explaining variance in condom use tendencies. This was done by a hierarchical multiple regression, where the percentage of variance in I-CUTE scores explained by barriers was tested, beyond the percentage explained by background variables, knowledge, and social desirability.

## Results

### Basic sample characteristics

Table 1 shows the study variables. Sample 1 included 60 (68.2%) men and 28 (31.8%) women. Approximately 50% had a partner. Sample 2 included 136 (65.1%) men and 73 (34.9%) women. Approximately 60% of the samples had no partner, while approximately 20% had a partner.

**Table 1.** Means and standard deviations (SD) and percentages of main study variables in samples one and two.

	Sample 1	Sample 2
Variable	Mean (SD)	Mean (SD)
Age	21.84 (2.38)	23.91 (3.63)
Gender		
Men, women	68.2, 31.8	65.1, 34.9
Having a partner		
No partner	50.6	59.8
Partner W.O. sexual relations		18.7
Several partners + sexual relations		3.8
One partner + sexual relations		17.7
I-CUTE	47.52 (7.67)	47.69 (7.59)
Condom use negotiation SE	15.12 (3.45)	
Social desirability	10.20 (3.01)	
Condom use barriers	65.03 (16.93)	62.50 (17.64)
Knowledge		2.12 (1.31)

#### Refining the I-CUTE

In sample 1, the internal reliability of the I-CUTE was Cronbach's Alpha = 0.74. In sample 2, the internal reliability of the I-CUTE was Cronbach's Alpha = 0.74. These values reflect adequate internal reliability.

#### Correlations between measures

Table 2 depicts the correlations between the I-CUTE scores and the main study variables in both samples. In sample 1, I-CUTE scores were significantly and positively correlated with the condom use negotiation self-efficacy scores. Moreover, the I-CUTE scores significantly and negatively correlated with the barriers scale scores. The I-CUTE had no significant correlation (only a trend) with social desirability scores. In sample 2, I-CUTE scores were again significantly and negatively correlated with the barriers scale scores. In contrast, knowledge test scores were unrelated to I-CUTE scores.

**Table 2.** Pearson correlations between condom use measures and social desirability.

	Sample 1	Sample 2
	I-CUTE	
CoN. SE	0.24*	
Barriers	−0.41**	−017*
SD	0.20	
Knowledge		0.02

Note: I-CUTE = indirect condom use test; CoN S.E = condom use negotiation self-efficacy; SD = social desirability.

*p < .05; **p < .01; ***p < .001.

### The contribution of barriers and knowledge to condom use tendencies

This analysis was conducted only in sample 2, where we assessed knowledge. We first examined the background correlates of the condom use tendencies. Among the background variables (age, gender, and having a partner), no variable was related to I-CUTE scores. Nevertheless, to be more rigorous, we considered all these variables in a multivariate regression. In this analysis, the first block of background variables (age, gender, having a partner) explained 0% of the variance in I-CUTE scores. In the second block, knowledge also explained 0% of the variance in I-CUTE scores as well. Finally, in the third block, condom use barriers explained an additional and significant 10.4% of the variance in I-CUTE scores, after statistically controlling for background variables and knowledge (*F* change (1,104) = 12.05, *p* = .001).

## Discussion

This study aimed to develop a new indirect instrument for assessing the tendency to use condoms, since this is a main protector against developing HIV (Smith et al., 2015; Weller & Davis-Beaty, 2002), but a socially highly sensitive topic. Given its sensitivity, its assessment via conventional self-reported tools is very problematic. Hence, we developed an indirect test, the I-CUTE. In addition, we aimed to examine the correlations between demographic, and background variables with the I-CUTE, and the contribution of barriers and knowledge to its scores.

The I-CUTE adopted the RPFT method of projection, but included preselected responses, to increase reliability and exclude interpretation biases by the investigator. The internal consistency of the 17-items I-CUTE was found to be adequate in two separate samples, European and African, supporting its reliability across cultures. In sample 1, I-CUTE scores were positively correlated with condom use negotiation self-efficacy, and negatively correlated with condom use barriers, supporting its convergent validity. Importantly, I-CUTE scores were not significantly correlated with social desirability scores, a finding which supports the divergent validity of the new test. In sample 2, I-CUTE scores were again negatively correlated with condom use barriers, supporting its convergent validity. This pattern of results supports our hypotheses that the I-CUTE correlates with other validated measures in a theoretically meaningful way. The fact that the I-CUTE scores are unrelated to social desirability supports our main aim to develop a scale that is not ‘contaminated’ by this factor. This is crucial because it is a socially sensitive issue, and it is important to estimate more accurately the use of condoms. This is particularly important in sub-Saharan Africa, where the prevalence of HIV is the highest in the world (Sam-Agudu, Folayan, & Ezeanulue, 2016), where the social norms and sensitivity concerning condom use may be especially important (Fladseth, Gafos, Newell, McGrath, & Hahn, 2015), yet is crucial because condom use is among the most effective methods for HIV prevention (Weller & Davis-Beaty, 2002). Beyond these crucial psychometric properties, the advantages of the I-CUTE include its ease of administration, its brevity and it being a user-friendly pictorial test.

Surprisingly, the knowledge test scores showed no correlation with the I-CUTE scores.

Furthermore, in a multiple regression, condom use barriers explained additional and significant variance in the indirect tendency to use condoms, beyond the effects of background measures and knowledge. This finding echoes those of past studies showing the importance of barriers in condom use (Mash et al., 2010) and extends them to an indirect measure of condom use. Finally, we found that barriers explain variance in the tendency to use condoms, independent of participants’ knowledge. The lack of contribution of knowledge also echoes results of a meta-analysis showing that condom use education has little or no significant effect on condom use (Gallant & Maticka-Tyndale, 2004). It is possible that one reason why educational programs have hardly increased condom use (Shaweno & Tekletsadik, 2013) is that they do not typically address people's social and internal barriers to condom use. Our results show the importance of such barriers in condom use, and suggest that future intervention studies will address such barriers, to possibly increase condom use. Once such emerging intervention is psychological inoculation (PI), which exposes participants to challenging sentences which reflect their social pressures and cognitive barriers, they must reject. Indeed, one pilot-controlled study found PI to have a stronger effect on condom use barriers than education alone (Olley, Abbas, & Gidron, 2011).

We phrased the items and the scenarios’ content primarily for female participants since past studies showed that women's personal factors (negotiation self-efficacy) were a more important predictor of condom use than that of men (Pulerwitz et al., 2002). Nevertheless, the reliability and correlation patterns of the I-CUTE with the other tested measures were similar in both genders (data not shown). This supports the use of this scale in both genders.

This study had several limitations. Firstly, the samples were convenience samples rather than representative ones. Nevertheless, our results showed consistency across several cultures. Secondly, we did not include any objective measure, such as number of purchased condoms, to validate the I-CUTE. However, this study's main aim was to develop a feasible and indirect measure of condom use, which bypasses presentation biases typically seen in self-reported questionnaires. Our results suggest that this was achieved by the non-significant correlation between the I-CUTE and social desirability. Future studies could replicate this validation in larger representative samples from diverse cultures, and in patients with and without HIV. Upon such cross-validation, this tool could be used for the evaluation of intervention trials aimed at increasing condom use toward reducing the risk of HIV, since persistent condom use reduces the risk of contracting HIV (e.g. Mansergh et al., 2015; Ramjee et al., 2015; Smith et al., 2015).

## Conclusions

This present study results demonstrate the reliability and validity of a new indirect measure for assessing the intentions to use condoms. These results were observed in two samples supporting the replicability of the findings. The positive correlations with condom use negotiation self-efficacy and negative correlations with condom use barriers, and lack of correlation with social desirability, demonstrate the convergent and discriminant validity of the I-CUTE. This tool can be used for evaluating the effectiveness of interventions aiming at increasing condom use, toward preventing HIV.
